# Personalized Information Service System of Smart Library Based on Multimedia Network Technology

**DOI:** 10.1155/2022/2856574

**Published:** 2022-09-07

**Authors:** Juan Wang

**Affiliations:** Library of Jiaxing University, Zhejiang Province, Jiaxing City 314001, China

## Abstract

Scientific and objective book quality evaluation and research on the value of books in the smart library are conducive to improving the reading needs of readers. However, it is difficult to obtain the important coefficient of the subjective and objective weight of the personalized information service index of the smart library at present, which has the problem of poor system performance. Therefore, a personalized information service system of the smart library based on multimedia network technology is designed. In multimedia network technology, the data collector module, data retriever module, and data memory module of the system hardware are designed. The Ethernet interface is connected through three buses to ensure the efficient operation of the system; according to the hardware, the software flow is introduced, which is divided into UI layer, logical business layer, and data access layer. On this basis, the application program is designed, all operation instructions follow the association rules, and the single-chip microcomputer is connected with the voice chip through the SPI serial port, so as to complete the design of the personalized information service system of the smart library. The experimental results show that the average absolute deviation of the designed system is small, the comprehensive performance is strong, and the update delay is kept within 0.6 s, which can improve the work efficiency.

## 1. Introduction

With the continuous development of society in recent years, the smart library, as the “concentration place” of books, is also the best place for readers to acquire knowledge and information in depth [1, 2]. Books make an important contribution to society and people's acquisition of cultural knowledge and are also an important product of human civilization and development [3, 4]. Titles were there was a substantial growth in recent years, at the same time there were many behind the rapid growth of performance driven by economic interests of hidden trouble, the main performance for the quality of books has plummeted, and similar content in the books published, and follow the repeat publishing phenomenon everywhere so that the books are of variable quality [5]. Due to the continuous development of information and communication technology and the wide application of the Internet, the personalized information service system management of books in smart libraries has become one of the main themes of the current library management reform and development [6, 7]. In this case, it is necessary to effectively design the personalized information service system in the smart library and establish a complete and scientific book quality evaluation index system in the smart library to identify the book quality in the smart library, so that more excellent books can stand out [8, 9].

Because the design method of the personalized information service system of the smart library has far-reaching development significance, it has also become a hot topic studied by experts and scholars, and has attracted extensive attention. Reference [10] proposes the role of media resource center in bringing novelty and creativity to Nigerian school library services. This article reveals the novelty of the functions of the center, especially in training qualified personnel to serve as librarians, media experts, reading teachers, and other school media personnel in Nigeria's preschool, primary, and postprimary education institutions, as well as organizing programmes related to children's reading and library use in Nigeria. Reference [11] proposes to evaluate the impact of traditional and digital marketing practices on university library services and resources. Determine how digital and traditional marketing methods can raise awareness among users and make better use of library services and resources. Understand the role of library staff in university library marketing practice and technology in digital and traditional ways. Based on quantitative research methods, cross-sectional survey research methods were used for data collection.

Although the above methods have made some progress, the application of multimedia network technology is not particularly sufficient, so the design of the intelligent library personalized information service system is based on multimedia network technology. Multimedia network technology refers to the text-based data communication and the text-based communication technology, including file transfer, e-mail, remote login, network news, and e-commerce. The technology is a comprehensive, interdisciplinary technology. It integrates the computer technology, network technology, communication technology, and a variety of information science technology achievements and has become the world's fastest developing and the most dynamic high-tech. In multimedia network technology, the data collector module, the data retrieval module, and the data storage module of the system hardware are designed, and the Ethernet interface is connected by three buses to ensure the efficient operation of the system. According to the hardware analysis software process, it is divided into UI layer, logical business layer, and data access layer. On this basis, the application program is designed, all operation instructions follow association rules, and the MCU is connected with the voice chip through the SPI serial port, thus the personalized information service system of the smart library is designed. The application in the personalized information service system of the intelligent library has remarkable effects, and the designed system has high performance.

## 2. Personalized Information Service System of Smart Library under Multimedia Network Technology

In multimedia network technology, the data collector module, data retrieval module, and data memory module of the system hardware are mainly designed, and the Ethernet interface is connected through three buses to ensure the efficient operation of the system; according to the hardware, the software process is introduced, which is divided into UI layer, logic business layer, and data access layer; on this basis, the application program is designed, all operation instructions follow the association rules, and the single-chip microcomputer and the voice chip are connected through the SPI serial port, thus completing the design of the personalized information service system of the smart library.

### 2.1. Hardware Design of the Personalized Information Service System of Smart Library

**Figure 1:**
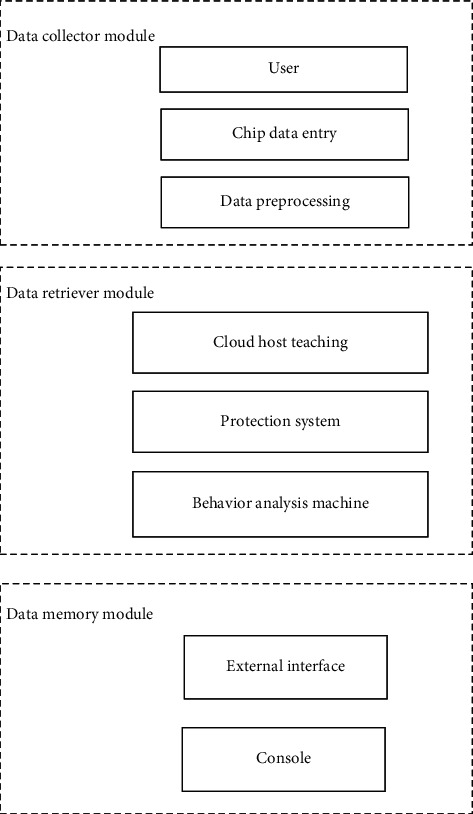
Hardware structure of the personalized information service system of smart library.

The basic components of multimedia network technology are: CD-ROM with optical drive, which is an important symbol of multimedia network technology system; It has the functions of A/D analog-to-digital conversion and D/A digital analog conversion, which can convert the analog signal and digital signal of voice to each other, so that the multimedia network technology has high-quality digital voice function; display with high definition [12, 13]. Under the multimedia network technology, the establishment of the personalized information service system of the smart library is the basis to ensure better service of resources. It can truly provide resources according to users' needs and enable users to obtain the richest resources, if allowed [14, 15]. The hardware function of the designed intelligent library personalized information service system is to use the client to feed back the data resources that customers need to retrieve and use the data collector module to collect library resources, and the data integrator will integrate and process the collected resources. The data searcher module will check and search the integrated data resources and finally feedback to the user by the data memory module [16]. The hardware structure of the personalized information service system of the smart library is shown in Figure 1.

The system hardware in Figure 1 applies multimedia network technology to share the resources of Smart Library. The system hardware adds a large number of embedded products to support various cloud classroom systems, improve teaching quality, and ensure students' learning efficiency [17].

#### 2.1.1. Data Collector Module

**Figure 2:**
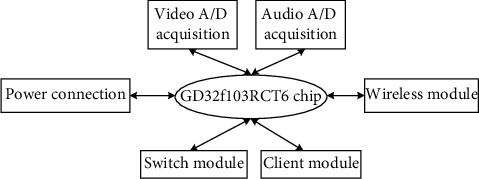
Collector structure.

The personalized information service system of the smart library designed in this paper can realize high-quality positioning collection, and compress the collected audio and video to meet the low-power requirements of the system [18, 19]. The internal chip of the collector is the GD32F103RCT6 chip, which was launched by Shenzhen Zhuocun Electronic Technology Co., Ltd. and has the characteristics of multifunctional multimedia application, which is convenient for collection, compression, and transportation [20, 21]. The GD32F103RCT6 chip consumes very low power. Generally, the chip can maintain normal acquisition work above 5 V voltage. The acquisition speed under low voltage can also reach 10000 MIPS, and the acquisition speed under high voltage is higher [22, 23]. The acquisition capability fully meets the acquisition requirements of large-scale library digital resources, and the acquisition accuracy is much higher than that of other chips, with an accuracy of 10 bits or even more [24]. The power supply mode of the data collector module is dual power supply of internal and external interfaces, and the internal interface is connected to the external interface to ensure the continuous input of voltage [25]. The collector is automatically connected to the wireless network. After signal coding and synthesis, it is transmitted to the wireless network data terminal, stored in the terminal, and recorded on the hard disk. The collector structure is shown in Figure 2.

When collecting video signals, the collector in Figure 2 selects SCLK as the clock to record all signals, and the input video is one video. When collecting GPS signals, data transmission signals, RX data reception signals, and asynchronous signal interfaces are used as power interfaces, and the maximum baud rate supported is 2.56 Mvyte/s. When collecting, it is necessary to connect and receive personalized information service system signals. The positioning accuracy of GPS signals is very strong, and the error rate is less than 4 m, which can locate high-speed mobile signals [26, 27]. The signal collector is connected by PCI and HPI, and the bus interface is an Ethernet interface. The transceiver can send and receive 10 M∼100 M personalized information service data. In order to realize the simultaneous transmission of service data network, a wireless module is added in the system.

#### 2.1.2. Data Retriever Module

The data searcher module is the core part of the hardware of the personalized information service system of the whole Smart Library. The searcher adopts the high-precision BAM6 chip developed by TRM Company in Norway. TRM fully analyzed the shortcomings and advantages of the previous two generations of BAM5 and BAM4 when developing the BAM6 chip. Therefore, this chip is significantly better than the previous two generations of chips in working time and retrieval efficiency [28]. The working delay time of the BAM6 chip is very short, only 10 minutes *μ*s. The rapid mode is used to control the retrieval speed, so the speed is very high. There are many retrieval interfaces inside. When the searcher runs, the interfaces will be connected together and work at the same time, which greatly reduces the working time, strengthens the retrieval ability, and retrieves more library digital resources [29]. The BAM6 chip requires the working voltage to be above 20 V and the data packet length of the chip is 45 B. It works under the multimedia network technology and the data retrieval rate is 80 Mb/s. However, in the low-voltage state, the chip will enter the automatic sleep mode and cannot start working.

#### 2.1.3. Data Memory Module

**Figure 3:**
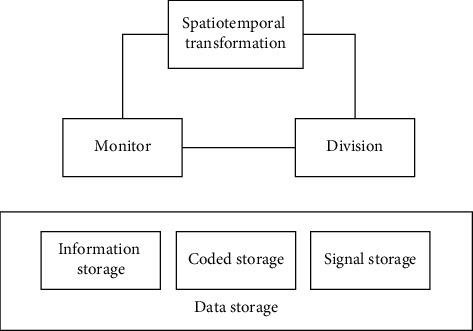
Memory structure.

In order to improve the storage efficiency of the hardware memory of the personalized information service system of the smart library, a flash memory with large storage range and low manufacturing cost is selected. A single-chip microcomputer is added to the memory to increase the storage capacity and reduce the floor area. The memory structure is shown in Figure 3.

There are three buses outside the memory, each bus is connected with an Ethernet interface, and different interfaces are connected with different signals [30]. The three wire buses are as follows:

  Bus 1: connect signals in I/O mode, input and output in two-way way, and realize two-way exchange of data. The remark mode is I/O.
  Bus 2: connect the signal in OUT mode, output the signal in one-way, and control the signal to enter.
  Bus 3: connect the signal in BUSY mode, and the input mode is busy signal input.

### 2.2. Software Design of the Personalized Information Service System of Smart Library

**Figure 4:**
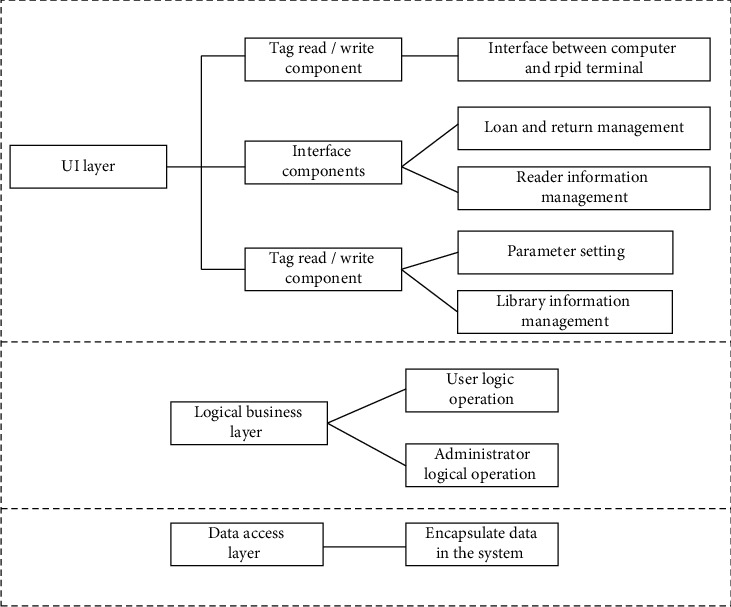
Software design of the personalized information service system of smart library.

In order to realize the software design of the intelligent library personalized information service system and complete the data receiving and processing, it communicates with the system terminal host computer through the wireless sensor network RS232 serial communication protocol of multimedia network technology. The gateway of the intelligent library personalized information service system uses socket process communication combined with a multithreading mechanism to complete the communication with the intelligent library personalized information service system terminal host computer [31]. The software structure of the personalized information service system of the smart library is shown in Figure 4.

As shown in Figure 4, the specific contents of the system software are as follows:

1. The UI layer of the intelligent library personalized information service system based on multimedia network technology, that is, the user interface of the intelligent library personalized information service system, is mainly used to realize the organic interaction between the intelligent library personalized information service system and readers, including the design of book borrowing, return, query interaction, reader information management, user interface attribute configuration, and so on. The UI layer is connected with the logical business layer (UI layer service providing layer). The UI layer of the intelligent library personalized information service system based on multimedia network technology designed in this paper includes two parts: RFID tag reading and writing component and user page component using the ISO/IEC14443 protocol. The RFID tag reading and writing component using the ISO/IEC14443 protocol is essentially the interface part between the PC and the RFID RF terminal to realize the data reading and writing function of the RFID RF terminal. The design of UI layer interface components mainly includes the book borrowing management module, reader information management module, book ID information management module, and system parameter attribute setting [32].
2. The logical business layer of the intelligent library personalized information service system based on multimedia network technology: the main function is all logical operation and processing of readers and managers.
3. Data access layer of the intelligent library personalized information service system based on multimedia network technology: it provides services for all logical operation and processing processes of readers and managers, and is mainly responsible for data access of the intelligent library personalized information service system database.

### 2.3. Application Design

The personalized information service system of the smart library connects the single-chip microcomputer with the voice chip through an SPI serial port, and all operation instructions must follow the association rules [33, 34]. All serial data transmission must be kept at a low level, and a high level should be used between two instructions. In order to ensure system security, a user authentication stage should be added in the process of application programming, and the system reminder function can be realized only through authorization.

In the implementation process, the layer-by-layer search iterative method is used to control the reminder function by calculating the confidence of association rules. The specific calculation contents are as follows:

(1) $$\begin{array}{c} D_{\text{abc}}=A_{a}\times B_{b}\times C_{c}. \end{array}$$

In formula (1), *A*_*a*_ represents the total amount of all data in the personalized information service data item set, *B*_*b*_ represents the total amount of all data in the item set contained in the smart library database, and *C*_*c*_ represents the total amount of support corresponding to each subserver.

In the application structure, the storage and playback of reminder instructions are controlled by pressing the key to realize the reminder function. Therefore, the elimination of key jitter and key response are introduced [35, 36]. The key jitter time is determined by the equipment performance. The software delay is used to detect whether the key is really pressed, which effectively avoids the key jitter time.

In order to reduce the error, set *F* to represent the end value of the counter, so as to obtain the key cycle as follows:

(2) $$\begin{array}{c} Y_{H}=\left(F-C_{s}\right)\times T_{z}. \end{array}$$

In formula (2), *C*_*s*_ represents the initial value of counting and *T*_*z*_ represents the clock cycle of a single-chip microcomputer. According to the key cycle shown in formula (2), the reminder time can be accurately mastered to avoid the delay problem of traditional system reminder.

When the system is started, the relevant reminder information is displayed on the display screen, and the prestored information is selected through the relevant keys of the keyboard. Under the multimedia network technology, the reminder instruction can be issued through the single-chip microcomputer controller of the hardware module to complete the design of the application program of the personalized information service system of the smart library.

## 3. Experimental Study

In order to test the actual working effect of the personalized information service system of the smart library based on multimedia network technology, an experiment is designed. The experimental environment is Intel(R) Pentium(R) 4, the CPU is 2.40 GHz, the memory is 1024 MB, and the operating system is Microsoft Windows XP SP3. Other experimental parameters are shown in Table 1.

According to the contents of Table 1, this paper uses the system in this paper, the system in reference [10] and the system in reference [11], respectively, to carry out the comparative experiment of intelligent library personalized information service system design. Three different methods are used to compare the accuracy of the personalized information service of the smart library. The average value of absolute deviation *P*_*JC*_ is used as the evaluation index of the accuracy of the personalized information service system. The average value of absolute deviation, i.e. the average deviation, refers to the average value of the absolute deviation of each measured value. The smaller the value, the better the effect of the personalized information service of the smart library. The calculation method is shown in

(3) $$\begin{array}{c} P_{\text{JC}}=\frac{\sum_{i\in S}\left|U_{i}-W_{i}\right|}{\left|S\right|}. \end{array}$$

**Figure 5:**
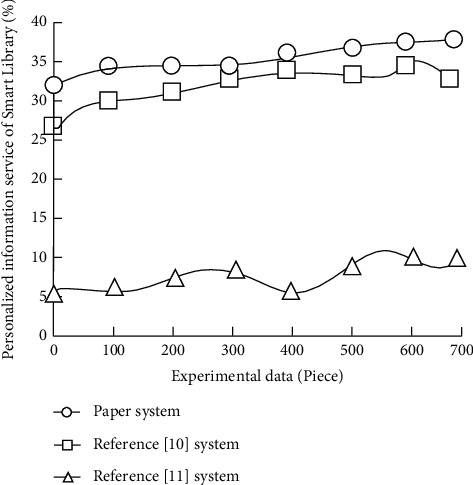
Comparison results of average absolute deviation of personalized information service of smart library under different methods.

In formula (3), |*S*| represents the book quality evaluation data set, *U*_*i*_ represents the actual scoring value of readers on item *i* Book personalized information service scheme in the book quality evaluation data set, and *W*_*i*_ represents the predicted scoring value of readers on item *i* scheme given by the book personalized information service system. Compare the average absolute deviation (%) of three different methods for personalized information service of the smart library, and the results are shown in Figure 5.

Through the analysis of Figure 5, it can be seen that the average absolute deviation of the personalized information service of the smart library using the system in this paper is lower than that of reference [10] system and reference [11] system. This is mainly because in the process of designing the personalized information service system of the smart library using the system in this paper, high-quality positioning acquisition can be realized, and the collected audio and video can be compressed. Get the important coefficient of subjective and objective weight of each evaluation index of the personalized information service in the smart library, so that the average absolute error of the personalized information service in the smart library using this system is low.

In order to further verify the effectiveness of the designed system, it is necessary to comprehensively analyze the effects of different performance of the system, and compare and analyze the storage performance, operation difficulty coefficient, expansion performance, security performance, and integration performance, respectively. The comparison results are shown in Table 2.

It can be seen from Table 2 that the storage performance of the system in this paper is good, the operation difficulty coefficient is small, the expansion performance and security performance are good, and it has the ability of data sharing and integration at the same time. While the storage performance of reference [10] system is general, the operation difficulty coefficient is large, the expansion performance is good, but the security performance is poor, it does not have data sharing, and the integration ability is also poor. Reference [11] system has general storage performance, large operation difficulty coefficient, and poor expansion performance, but it has good security, data sharing, and general integration ability. Based on the traditional system, the designed system improves the storage performance of the system to make the storage performance of the designed system better.

**Figure 6:**
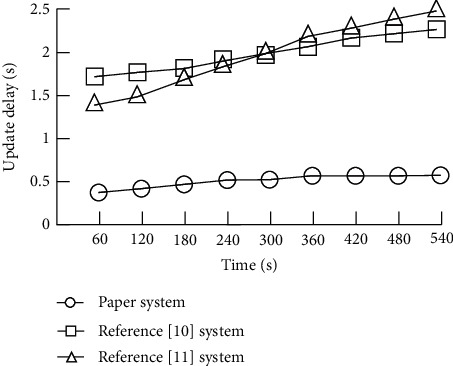
Book information update delay of the personalized information service system of smart library.

Continue to explore the effects of the personalized information service system of the smart library based on multimedia network technology. The working frequency of the system is detected every 60 s, and there is no waiting time when readers query relevant book information. The time difference between the actual database update frequency and the corresponding book information update frequency of the designed system is defined as the update delay (s). The comparison results are shown in Figure 6.

According to Figure 6, compared with reference [10] system and reference [11] system, the book information refresh frequency of the personalized information service system of the smart library based on multimedia network technology designed in this paper can meet the practical requirements of the smart library. The system update delay is kept within 0.6 s, which can effectively realize multiple links such as on-board book inventory, return to the shelf, and query circulation, and greatly improve the work efficiency of the system.

To sum up, the designed system has good performance and realizes the intelligent inventory function and personalized service of personalized information service of the University Smart Library. With the help of the application of the personalized information service system of the University Smart Library, the work progress of the library and the work efficiency of librarians are greatly improved, which can better serve readers.

## 4. Discussion and Analysis

In terms of multimedia network technology, aiming at the development of personalized information service of the smart library, the countermeasures are as follows:

### 4.1. Improving the Management Mechanism of Personalized Information Service

Due to the lack of a macro-control mechanism in China's libraries, in order to coordinate and develop libraries and realize the common knowledge and sharing of information resources, China's higher education document guarantee system has been established. Strengthen the cooperation between the system and its member museums, improve the service level of higher education, and give full play to the maximum social and economic benefits. Strengthening the service consciousness and management means alone cannot meet the requirements of improving the service quality of the library. The cooperation between departments, the adjustment of structure, and the unification of the evaluation system are indispensable. Master the information needs of users, track and evaluate the service quality, pay attention to the feedback of user satisfaction, adopt incentive mechanism to stimulate the initiative of service personnel, and provide users with comprehensive and high-quality personalized information services. To develop the library personalized information service, the improvement of library personalized information service mechanism plays a very important role in the development of the smart library.

### 4.2. Strengthening the Construction of Information Service Resources

#### 4.2.1. Strengthening the Content Construction of Information Resources

Establish a professional navigation database and a characteristic database. In order to cope with the rapid expansion and disorder of network information resources, the smart library should collect, sort, and classify the network information resources according to the specialty according to the characteristics of teachers and students, for the purpose of meeting users' learning and scientific research, highlight the professionalism of subject navigation, and establish professional navigation links. For the construction of information resources, the smart library is based on the careful investigation of databases at home and abroad. According to the professional setting of the university, the focus of scientific research and the information needs of users, combined with the library's collection resources, the smart library establishes a characteristic database according to a certain discipline, specialty, or local characteristics.

#### 4.2.2. Strengthening the Organization and Integration of Information Resources

Resource integration and service integration are the main contents of information resource integration. Resource integration refers to the integrated information system formed by classifying and sorting the existing information resources. Service integration needs to build a personal information database, which includes the basic information of users and feedback information of users on services. Compared with traditional information services, in the organization of information resources, the new requirements of personalized information services are: the content should be targeted, clear, easy to understand, open, and flexible; in the navigation system, the classification should be detailed and reasonable; the user interface should be friendly; the evaluation ability and information navigation should be strong; and the cross-platform seamless connection of information resource content should be realized. The library needs to sort out and classify the discrete electronic resources and establish a professional navigation database with a friendly interface, powerful functions, and comprehensive according to the needs of users.

#### 4.2.3. Strengthening the Co-Construction and Sharing of Information Resources

In the school, each department will establish its own library reference room according to its own situation, which overlaps with the resource construction of the library, resulting in a low utilization rate and a waste of material and human resources. Therefore, strengthening the cooperation between reference room and library and jointly building and sharing information resources in the school is the main measure of information co-construction and sharing. Smart libraries can give full play to their respective advantages of document resources, which not only avoids the repeated construction of resources but also enriches the acquisition of users' information resources.

### 4.3. Changing the Service Concept and Improving the Quality of Service Personnel

The establishment of a humanistic care service concept of “people-oriented, user-first” is very beneficial to the active and effective development of personalized information services in the library. “People-oriented and service-oriented” refers to putting the needs of users in the first place, taking the completion of this goal as the fundamental purpose of the work, all for the sake of users and convenience, and providing users with maximized and optimized services. To meet the service needs of users, service personnel should have strong information analysis ability and language ability to answer various questions for users; we should broaden the scope of knowledge and strengthen learning, especially the relevant knowledge of library and information; master skilled information skills; be able to analyze, integrate, and summarize information; and master the sources and retrieval methods of various types of information. Only with these can service personnel improve the service level and provide users with obvious personalized information services. “Subject Librarian” is a kind of special personalized information service talent arising from the problems of low technical level, single knowledge, and poor information analysis ability of library service personnel. Subject librarian refers to the person that the library sets up to establish contact with a certain department or discipline specialty as the counterpart unit, build a bridge between the department, discipline specialty, and the library, communicate with each other, and actively collect and provide document information services for users. This kind of service personnel needs to have a certain level of foreign language, be familiar with library business, be skilled in computer operation, have high educational background and professional title, profound cultural heritage and strong language ability, and be able to provide powerful help for teaching and scientific research.

### 4.4. Strengthening the Construction of Personalized Information Service Systems

To develop a personalized information service system, the library should have both rich system resources and rich system functions, and be able to provide comprehensive services. The designed system interface should be concise, intuitive, and clear-cut, and users should be able to make personalized customizations; it should be able to realize automatic integration with other resource systems, which not only saves users' time but also reduces users' use burden; and can protect user privacy. The measures to improve the personalized information service include the following: establishing a professional navigation system, building an effective information space, systematically organizing relevant information resources, providing users with a good retrieval interface, information customization, and information push. The system can provide users with selection services. Users can also select and manage information to realize their interactive functions. The problems encountered in use can be solved in time to improve the service quality.

To sum up, under the multimedia network technology, the smart library will encounter many problems in the process of building personalized information services. The smart library needs to improve its personalized service management mechanism, strengthen the construction of information resources, strengthen the construction of personalized information service system, and strengthen the research and training of user needs. While changing the service concept, library service personnel should also pay attention to the improvement of their own quality, improve the service quality, and make the library's personalized information service develop continuously.

## 5. Conclusions and Prospects

### 5.1. Conclusions

1. The average absolute deviation of the intelligent library personalized information service system based on multimedia network technology is lower than that of the reference system, which has a good effect. It can realize high-quality location collection and get the important coefficient of subjective and objective weight of each evaluation index of personalized information service a in smart library.
2. The system in this paper has good storage performance, low operation difficulty coefficient, good expansion performance, and security performance, as well as data sharing and integration ability.
3. The book information refresh frequency of the personalized information service system designed in this paper can meet the practical requirements of the smart library, and the system update delay is kept within 0.6 s, which effectively improves the working efficiency of the system. With the application of the personalized information service system of the university intelligent library, the progress of library work and the work efficiency of librarians have been greatly improved, and it can serve readers better.

### 5.2. Prospects

The personalized information service system of the smart library realizes the unattended of the library, uses the accurate positioning of multimedia network technology and the realization of self-help borrowing and returning books, reduces the labor intensity of the staff, and enables them to devote more energy to scientific research. However, in order to serve college teachers and students more perfectly, there are still a lot of problems to be solved in order to explore a more intelligent, personalized information service mode.

The further development of the personalized information service system of the smart library can use the mobile device library platform for subsequent docking on the basis of this information service system and realize a more efficient and intelligent library information service system through the construction of the mobile platform. For example, it can realize that the mobile device can receive an update of all library information, the system can automatically urge the return of books, etc. Interlibrary communication and information sharing can be achieved by relying on the mobile platform to improve the quality of interlibrary communication and information sharing. Relying on the advantages of multimedia network technology, we can popularize the installation and popularization scope of book borrowing and returning terminals, open library resources, break the constraints of time and space, and realize the possibility of systematic remote management and real-time circulation of books through the push of terminal points, so as to improve the circulation times in the book cycle.

The development of technology and the popularization of networks promote the development of library cause. In multimedia network technology, the personalized information service of the Smart Library continues to develop and progress with various changes. The specific contents are as follows:

1. Under the network environment, the information service of smart library is gradually open to the society from the closed state that only faces the teachers and students of the University. Each smart library will no longer “act in its own way,” but build a network information platform and share information resources through networking.
2. The smart library is constantly changing its service mode due to the change of users' needs, changing from the traditional passive service mode to an active service, actively understanding the new trends of users, and adjusting the service content in time.
3. The service personnel of smart library are aware of the shortcomings of the traditional service concept, actively change their own service concept, and gradually establish a new concept of “people-oriented, user-first.”
4. The knowledge cultivation and ability of service personnel in Smart Library are constantly improving to adapt to the continuous development of personalized information service. In particular, the establishment of “Subject Librarian” is a prominent embodiment of talent training in personalized information service.
5. The strengthening of the awareness of training users by the smart library enables more users to master the ways and means of obtaining information and master the retrieval skills and tools, which is conducive to users to make better use of various resources in the library and improve the utilization rate of library information resources.

## Figures and Tables

**Table 1:** Experimental parameter setting table.

Serial number	Entry name	Parameter content
1	Transmission signal	Audio signal, video signal, and GPS signal
2	Data line distance	1500 m
3	Input interface	TUB interface
4	Output interface	VUTR interface
5	Resolving power	One hundred and sixty × 128 dpi
6	Operating hours	Usually 24 h
7	Power consumption	<1 W

**Table 2:** Comparison of different performance of different platforms.

Performance	Paper system	Reference [10] system	Reference [11] system
Storage performance	Preferably	Commonly	Commonly
Operation difficulty coefficient	Less	More	More
Extended performance	Preferably	Good	Poor
Safety performance	Preferably	Difference	Preferably
Data sharing	Have	No	Have
Integration performance	Preferably	Poor	Commonly

## Data Availability

The raw data supporting the conclusions of this article will be made available by the author without undue reservation.
